# Association of High Levels of Antidrug Antibodies Against Atezolizumab With Clinical Outcomes and T-Cell Responses in Patients With Hepatocellular Carcinoma

**DOI:** 10.1001/jamaoncol.2022.4733

**Published:** 2022-10-20

**Authors:** Chan Kim, Hannah Yang, Ilhwan Kim, Beodeul Kang, Hyeyeong Kim, Hyunho Kim, Won Suk Lee, Sanghoon Jung, Ho Yeong Lim, Jaekyung Cheon, Hong Jae Chon

**Affiliations:** 1 Medical Oncology, Department of Internal Medicine, CHA Bundang Medical Center, CHA University School of Medicine, Seongnam, Korea; 2 Division of Oncology, Department of Internal Medicine, Inje University College of Medicine, Haeundae Paik Hospital, Busan, Korea; 3 Department of Internal Medicine, Ulsan University Hospital, University of Ulsan College of Medicine, Ulsan, Korea; 4 Division of Medical Oncology, Department of Internal Medicine, St Vincent’s Hospital, College of Medicine, The Catholic University of Korea, Suwon, Korea; 5 Department of Radiology, CHA Bundang Medical Center, Seongnam, Korea; 6 Department of Internal Medicine, Samsung Medical Center, Sungkyunkwan University, Seoul, Korea

## Abstract

**Importance:**

Administration of atezolizumab could be immunogenic and induce undesirable antidrug antibody (ADA) responses. This may interfere with atezolizumab-mediated actions, affecting drug clearance and serum concentration or inducing antibody neutralization.

**Objective:**

To determine the clinical and immunological associations of highly elevated ADA levels with clinical outcomes after atezolizumab/bevacizumab (Atezo/Bev) treatment in patients with advanced hepatocellular carcinoma (HCC).

**Design, Setting, and Participants:**

This cohort study prospectively enrolled 174 patients with advanced HCC treated with first-line Atezo/Bev (discovery cohort: 61 patients from 1 center; validation cohort: 113 patients from 4 centers).

**Exposures:**

Serum ADA levels at pretreatment and 3 weeks (cycle 2 day 1 [C2D1]) were analyzed using competitive enzyme-linked immunosorbent assays. In addition, samples were subjected to serological and flow cytometric analyses.

**Main Outcomes and Measures:**

Overall, ADA positivity was associated with treatment outcomes and T-cell functions.

**Results:**

After excluding patients with inadequate samples, follow-up loss, or consent withdrawal, 132 patients (discovery cohort: 50 patients; 41 [82.0%] men; median age [IQR], 61 [55-70] years; validation cohort: 82 patients; 70 [85.4%] men; median age [IQR], 61 [53-68] years) were analyzed, and robust ADA (≥1000 ng/mL) responses at C2D1 were identified in 23 (17.4%) of the patients. Patients with progressive disease exhibited higher ADA levels (median [IQR], 65.2 [0-520.4] ng/mL) at C2D1 than in responders (median [IQR], 0 [0-117.5] ng/mL). In both discovery and validation cohorts, patients with high ADA levels at C2D1 were associated with a reduced response rate (discovery cohort: 34% vs 11%; validation cohort: 29% vs. 7%) and worse progression-free survival (discovery cohort: hazard ratio [HR], 2.84; 95% CI, 1.31-6.13; *P* = .005; validation cohort: HR, 2.52; 95% CI, 1.27-5.01; *P* = .006) and overall survival (discovery cohort: HR, 3.30; 95% CI, 1.43-7.64; *P* = .003; validation cohort: HR, 5.81, 95% CI, 2.70-12.50; *P* = .001) with Atezo/Bev compared with those with low ADA levels. In multivariable Cox regression, the clinical implication of high ADA levels persisted even after adjusting for various confounding factors and was most significant at 1000 ng/mL or greater. Compared with patients with low ADA levels, patients with high ADA levels exhibited reduced serum atezolizumab concentrations, impaired CD8-positive T-cell proliferation, and had decreased interferon-γ and tumor necrosis factor-α from CD8-positive T cells compared with patients with low ADA levels.

**Conclusions and Relevance:**

This cohort study found that highly elevated ADA levels at C2D1 may be associated with poor clinical outcomes in patients with advanced HCC treated with Atezo/Bev. High ADA levels may reduce atezolizumab exposure and attenuate the anticancer efficacy of the drug.

## Key Points

**Question:**

What are the clinical and immunologic associations of atezolizumab antidrug antibody (ADA) levels with clinical outcomes after atezolizumab/bevacizumab treatment in patients with advanced hepatocellular carcinoma (HCC)?

**Findings:**

In this cohort study examining 132 patients with advanced HCC, highly elevated ADA levels (≥1000 ng/mL) at 3 weeks were associated with poor clinical outcomes. Compared with patients with low ADA levels, patients with high ADA levels exhibited reduced systemic exposure to atezolizumab and impaired proliferation and activation of peripheral CD8-positive T cells.

**Meaning:**

Developing high ADA levels at an early time point could attenuate the immunotherapeutic efficacy of atezolizumab.

## Teaser

This cohort study examines the clinical and immunologic associations of atezolizumab antidrug antibody levels with patient outcomes after treatment with atezolizumab/bevacizumab in patients with advanced hepatocellular carcinoma.

## Introduction

Combination therapy with atezolizumab and bevacizumab (Atezo/Bev) in the IMbrave 150 trial has dramatically altered the treatment landscape of advanced hepatocellular carcinoma (HCC).^1,2,3^ Although patients who responded to Atezo/Bev therapy showed favorable survival outcomes, a fraction of patients still exhibited primary resistance.^4,5,6^

Administration of immune checkpoint inhibitors (ICIs) can be immunogenic and induce undesirable antidrug antibody (ADA) responses.^7,8,9,10,11^ These ADAs can interfere with the functions of therapeutic antibodies, affecting drug clearance and serum concentration, or even inducing antibody neutralization.^7,8,9,12^ In the IMbrave 150 study,^13^ 29.6% of patients with advanced HCC developed atezolizumab ADAs following Atezo/Bev treatment. However, data are lacking regarding the pattern of ADA development outside the clinical trial setting or to guide treatment decisions in patients with HCC receiving Atezo/Bev therapy.

Herein, we elucidated the clinical and immunologic associations of highly elevated ADA levels with outcomes at 3 weeks after Atezo/Bev treatment (cycle 2 day 1 [C2D1]) in patients with advanced HCC.

## Methods

The present study was conducted in 2 stages. In the discovery cohort, patients with HCC treated with Atezo/Bev were prospectively enrolled at the CHA Bundang Medical Center. For the validation cohort, patient enrollment was extended to 4 tertiary cancer centers in Korea (CHA Bundang Medical Center, Ulsan University Hospital, Haeundae Paik Hospital, and St Vincent Hospital). The study was approved by relevant institutional review boards and all patients provided written informed consent.

The eligibility criteria were age 20 years or older, locally advanced or unresectable HCC confirmed by histologic or cytologic analysis, or clinical features according to the American Association for the Study of Liver Diseases criteria for patients with cirrhosis, no prior systemic therapy, Child-Pugh class A, and an Eastern Cooperative Oncology Group (ECOG) performance status of 0 to 1.

Blood samples were collected before the first administration of atezolizumab (cycle 1 day 1, hereafter referred to as baseline) and before the second atezolizumab injection (C2D1). The C2D1 blood samples drawn 18 days before or more than 24 days after treatment initiation were excluded from the analysis to ensure consistent timing.

Serum atezolizumab (C2D1) and ADA (baseline and C2D1) levels were analyzed using enzyme-linked immunosorbent assays (KBI1027 and KBI2027, respectively; KRISHGEN BioSystems). Multiplex flow cytometric analysis was used to examine the association of ADA with T-cell immunity.

## Results

From June 2020 to July 2021, we prospectively enrolled 174 patients with advanced HCC treated with first-line Atezo/Bev in the discovery and validation cohorts (discovery cohort: 61 patients from a single center; validation cohort: 113 patients from 4 centers). After excluding 42 patients with inadequate samples, follow-up loss, or consent withdrawal, ADA responses were analyzed in 132 patients (discovery cohort: 50 patients; validation cohort: 82 patients) (eFigure 1 in the Supplement). The Table presents the baseline characteristics of patients in the discovery and validation cohorts. The discovery and validation cohorts had a median follow-up of 19.4 and 13.4 months, respectively.

**Table.** Baseline Demographics of Patients With Hepatocellular Carcinoma

Characteristic	Cohort, No. (%)		
	Total HCC	Discovery	Validation cohort
No.	132	50	82
Age, median (IQR)	61 (55-69)	61 (55-70)	61 (53-68)
Male sex	111 (84.1)	41 (82.0)	70 (85.4)
ECOG performance status			
0	71 (53.8)	25 (50.0)	46 (56.1)
1	61 (46.2)	25 (50.0)	36 (43.9)
Child-Pugh classification			
A5	86 (65.2)	35 (70.0)	51 (62.2)
A6	46 (34.8)	15 (30.0)	31 (37.8)
Barcelona Clinical liver cancer stage			
B	24 (18.2)	10 (20.0)	14 (17.1)
C	108 (81.8)	40 (80.0)	68 (82.9)
Alpha-fetoprotein ≥400 ng/mL	42 (31.8)	16 (32.0)	26 (31.7)
Neutrophil to lymphocyte ratio, median (IQR)	2.6 (1.7-4.2)	2.7 (1.6-4.4)	2.6 (1.8-4.0)
Presence of macrovascular invasion	55 (41.7)	20 (40.0)	35 (42.7)
Presence of extrahepatic spread	74 (56.1)	28 (56.0)	46 (56.1)
Etiology of HCC			
Hepatitis B	89 (67.4)	34 (68.0)	55 (67.1)
Hepatitis C	7 (5.3)	4 (8.0)	3 (3.7)
Alcohol	21 (15.9)	8 (16.0)	13 (15.9)
Other or unknown	15 (11.4)	4 (8.0)	11 (13.4)
Prior local therapy for HCC	81 (61.4)	34 (68.0)	47 (57.3)
ADA, median (IQR), ng/mL			
At baseline	0	0	0
At C2D1	45.95 (0-257.9)	77.45 (0-356.0)	33.55 (0-193.1)
Antidrug antibody levels at C2D1			
Negative or low (<1000 ng/mL)	109 (82.6)	41 (82.0)	68 (82.9)
High (≥1000 ng/mL)	23 (17.4)	9 (18.0)	14 (17.1)

Abbreviations: ADA, antidrug antibody; CTD1, cycle 2 day 1 of treatment; ECOG, Eastern Cooperative Oncology Group; HCC, hepatocellular carcinoma.

Compared with baseline levels, atezolizumab ADA levels were elevated at C2D1 (median [IQR] 0 [0-0] vs. 45.95 [0-257.9] ng/mL; *P*<.001) (eFigure 2A in the Supplement). Although most patients did not develop atezolizumab ADAs or exhibited very low ADA levels at C2D1, a fraction of patients presented a very rapid and robust ADA response at C2D1. Participant ADA levels at C2D1 were markedly higher in patients with progressive disease than in those with a complete response/partial response or stable disease (discovery cohort: median, 265.05 ng/mL vs. 0 ng/mL; *P* = .008; validation cohort: median, 62.6 vs. 0 ng/mL; *P *= .01). (eFigure 2B in the Supplement). To focus on the posttreatment ADA response and to minimize false-positive results, we set 1000 ng/mL as the ADA cutoff for grouping patients as ADA high (≥1000 ng/mL) or ADA low (<1000 ng/mL). Based on this cutoff value, 23 (17.4%) patients with advanced HCC treated with Atezo/Bev exhibited high ADA levels at C2D1 (Table).

**Figure 1.**
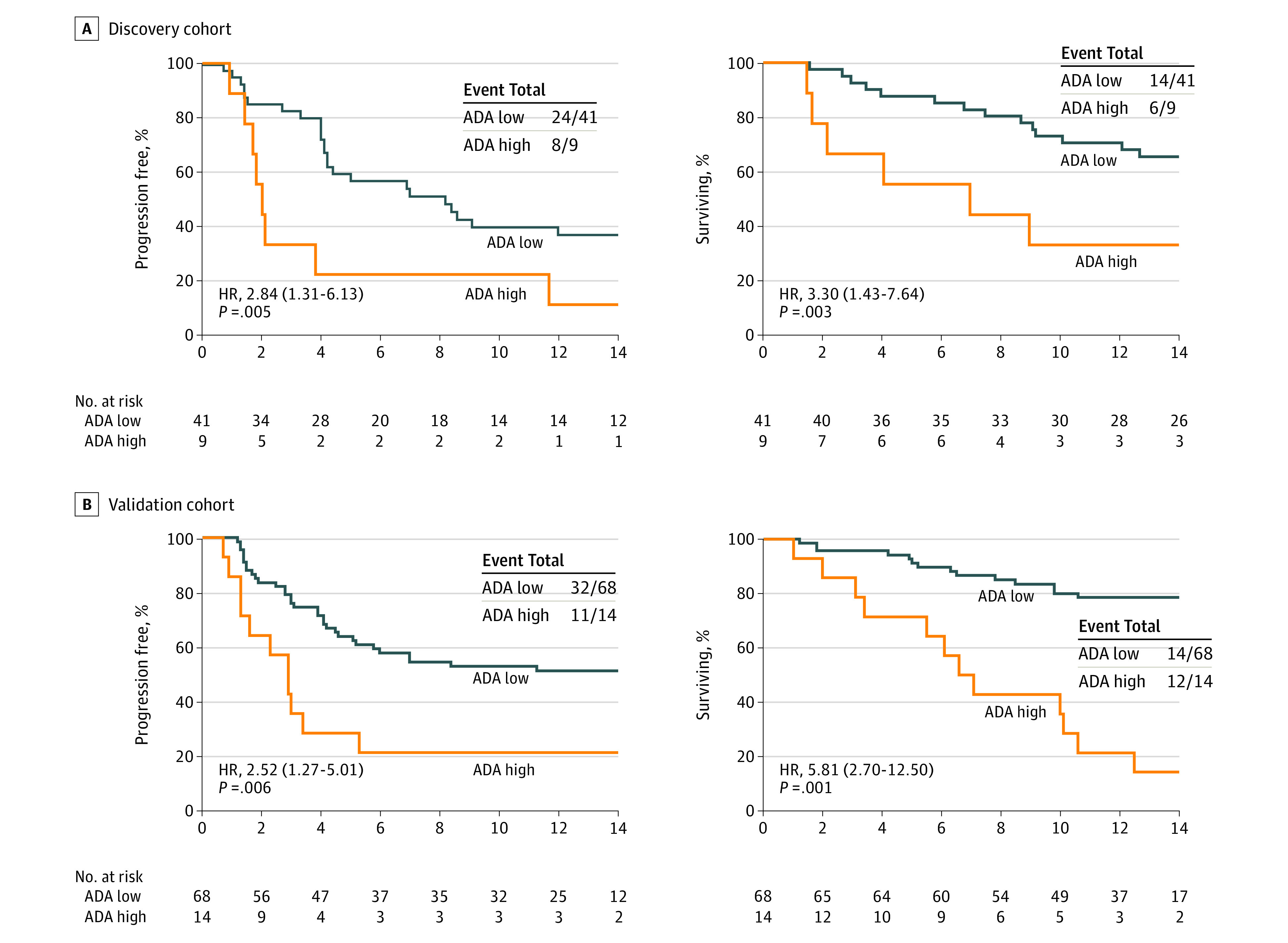
Progression-Free Survival and Overall Survival According to Antidrug Antibody Status

Next, we compared the clinical outcomes of Atezo/Bev according to the ADA status at C2D1. In both discovery and validation cohorts, patients with high ADA levels at C2D1 showed a decreased response rate (eFigure 3 in the Supplement) and shorter progression-free survival (PFS) and overall survival (OS) with Atezo/Bev compared with those with low ADA levels (Figure 1). Overall, ADA levels of 1000 ng/mL or greater had a favorable predictive value for OS in the time-dependent receiver operating characteristic curve (area under curve, 0.78; 95% CI, 0.63-0.82) (eFigure 4 in the Supplement).

In the multivariable analysis with discovery and validation cohorts, high ADA levels remained independently associated with shorter PFS and OS even after adjusting for age, sex, ECOG performance status, Child-Pugh score, α-fetoprotein, macroscopic vascular invasion, extrahepatic spread, and neutrophil-to-lymphocyte ratio (eFigure 5A in the Supplement). Notably, lowering the cutoff value reduced the unfavorable clinical effect of ADA on PFS and OS (eFigure 5B in the Supplement).

**Figure 2.**
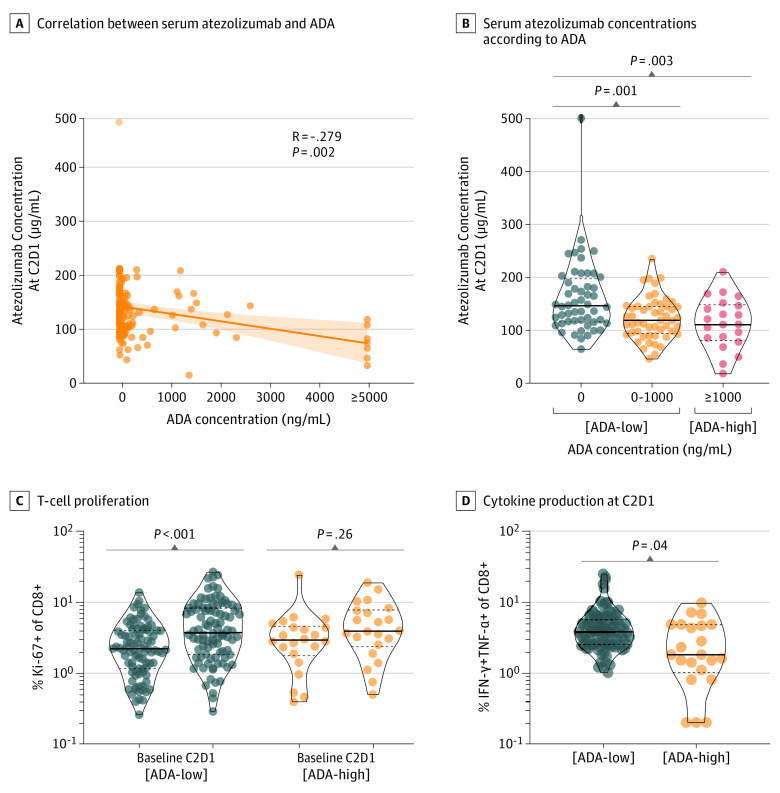
Serum Atezolizumab Concentration and T-Cell Functions According to Antidrug Antibody (ADA) Status A, Correlations between serum atezolizumab and ADA concentration. B, Comparisons of serum atezolizumab concentrations according to ADA level. C, Ki67-positive proliferating CD8-positive T cells between baseline and C2D1 according to ADA status. D, Interferon-γ and tumor necrosis factor-α from CD8-positive T cells according to ADA status.

To further elucidate how high ADA levels affect the immunotherapeutic efficacy of atezolizumab, we evaluated atezolizumab concentrations and T-cell phenotypes according to ADA status. The concentration of atezolizumab at C2D1 was inversely correlated with ADA levels, and atezolizumab concentration at C2D1 significantly decreased in patients with ADA values of 1000 ng/mL or greater (Figure 2, A and B). The fraction of Ki-67– andCD8- positive proliferating T cells was remarkably increased at C2D1 in the low-ADA group; however, this fraction was not significantly altered in the high-ADA group (Figure 2C). Moreover, the high-ADA group showed reduced interferon-γ and tumor necrosis factor-α from CD8-positive T cells compared with the low-ADA group (Figure 2D).

## Discussion

Herein, we observed that 23 (17.4%) patients with advanced HCC developed robust ADA responses 3 weeks after initiating Atezo/Bev treatment (C2D1), along with unfavorable survival outcomes. These findings were consistent with those of the discovery and validation cohorts. High ADA levels were clinically significant even after adjusting for various confounding factors. Furthermore, we elucidated the biological association of high ADA levels with atezolizumab concentration and CD8-positive T-cell proliferation and function. These findings suggest that developing high ADA levels at an early time point may attenuate the immunotherapeutic efficacy of atezolizumab.

Although previous ADA studies were conducted by sponsors using samples from sponsor-initiated clinical trials,^13,14^ this prospective study was investigator initiated and demonstrated the clinical and immunological association of atezolizumab with ADAs in patients with HCC.

To define a clinically relevant ADA level, we established a sufficiently high cutoff value to define ADA positivity. Although a high ADA level of 1000 ng/mL or greater could precisely predict PFS and OS with Atezo/Bev treatment, we noted that the statistical significance gradually declined as the ADA cutoff level decreased. Accordingly, the atezolizumab concentration at C2D1 was inversely correlated with ADA levels.

### Limitations

This study enrolled a limited number of East Asian (Korean) patients in an endemic region of hepatitis B virus (HBV). Therefore, it is necessary to confirm this in a larger number of patients, including other ethnic groups. Moreover, this study focused on ADA levels at 3 weeks, an early time point, but did not evaluate the prevalence of neutralizing antibody, which is a relatively later event. Furthermore, a cutoff point for ADA positivity should be optimized and validated in future studies. To address these limitations, another study (NCT05173298) is ongoing.

## Conclusions

This cohort study found that highly elevated ADA levels (≥1000 ng/mL) at 3 weeks (C2D1) may be associated with poor clinical outcomes in patients with advanced HCC treated with Atezo/Bev. Further validation and standardization of ADA assays are warranted to optimize atezolizumab-based immunotherapy.

## Notes

Supplement. **eFigure 1.** CONSORT diagram **eFigure 2.** Serum atezolizumab ADA levels **eFigure 3.** Bar charts showing the best response to therapy by ADA status **eFigure 4.** A time-dependent receiver operating characteristic curve for 12-months OS **eFigure 5.** Multivariable analysis of PFS and OS according to ADA status
eMethods
