# Supplementary material for: Association of High Levels of Antidrug Antibodies Against Atezolizumab With Clinical Outcomes and T-Cell Responses in Patients With Hepatocellular Carcinoma
Source: JAMA Oncol. 2022 Oct 20;8(12):1825–9. doi: 10.1001/jamaoncol.2022.4733 (PMC9585463; doi:10.1001/jamaoncol.2022.4733)
Supplement: Supplement. — eFigure 1. CONSORT diagram eFigure 2. Serum atezolizumab ADA levels eFigure 3. Bar charts showing the best response to therapy by ADA status eFigure 4. A time-dependent receiver operating characteristic curve for 12-months OS eFigure 5. Multivariable analysis of PFS and OS according to ADA status eMethods [file jamaoncol-e224733-s001.pdf]

Supplemental Online Content

Kim C, Yang H, Kim I, Kang B, Kim H, et al. Association of high levels of antidrug antibodies against atezolizumab with clinical outcomes and T-cell responses in patients with hepatocellular carcinoma. *JAMA Oncol*. Published online October 20, 2022. doi:10.1001/jamaoncol.2022.4733

eFigure 1. CONSORT diagram

eFigure 2. Serum atezolizumab ADA levels

eFigure 3. Bar charts showing the best response to therapy by ADA status

eFigure 4. A time-dependent receiver operating characteristic curve for 12-months OS

eFigure 5. Multivariable analysis of PFS and OS according to ADA status

eMethods

This supplemental material has been provided by the authors to give readers additional information about their work.

© Kim C, et al. *JAMA Oncol*. 2022.

eFigure 1. CONSORT diagram

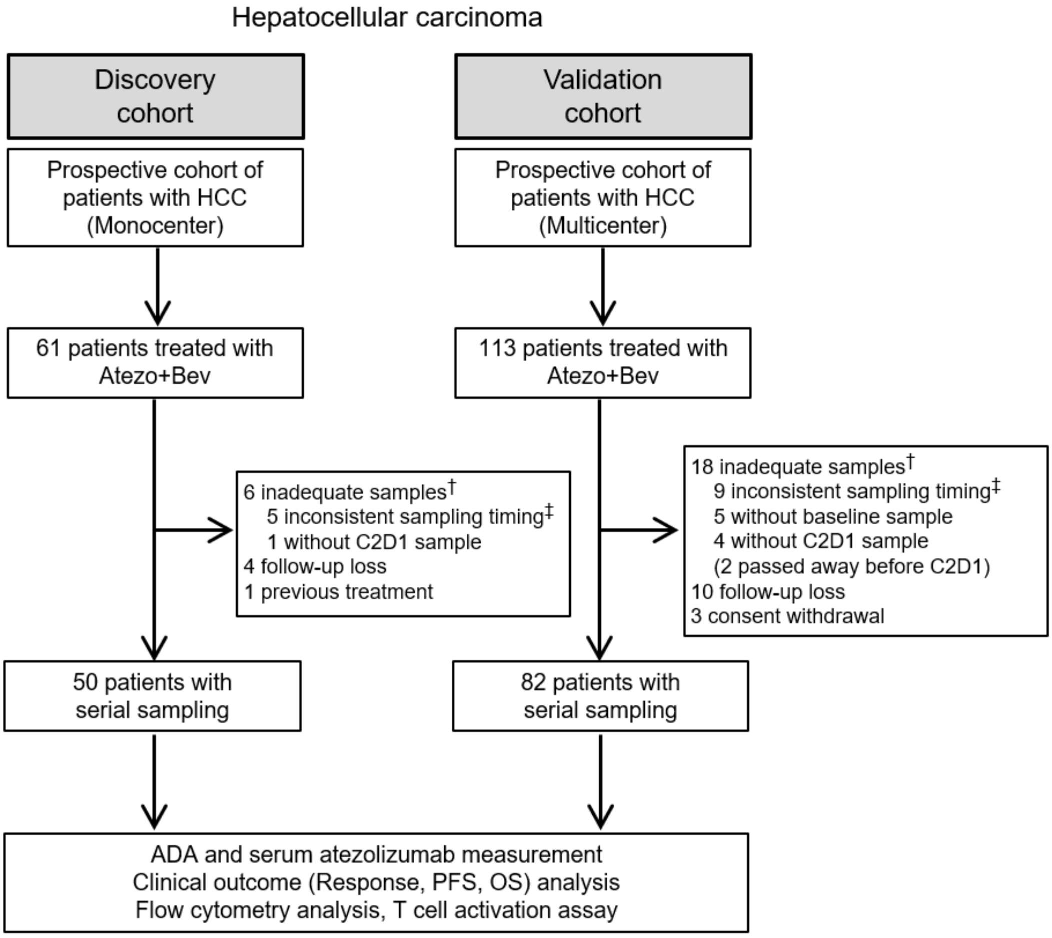

†54.5% (6/11) of the excluded/unavailable samples in the validation cohort and 51.6% (16/31) of the excluded/unavailable samples in the discovery cohort were related to COVID-19 pandemic issues (lockdowns, quarantines of patients, and delays in logistic systems).

‡C2D1 blood samples that were drawn before 18 days or more than 24 days after the initiation of the first treatment were excluded from the analysis to ensure consistent timing.

© Kim C, et al. *JAMA Oncol*. 2022.

eFigure 2. Serum atezolizumab ADA levels

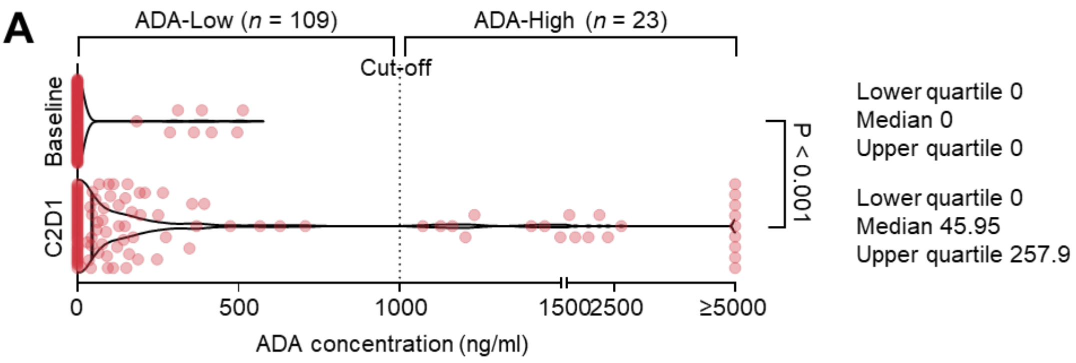

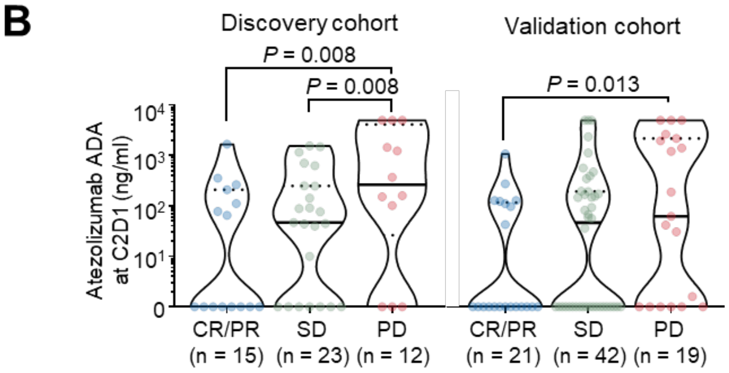

(A) Pooled analysis of ADA levels at baseline and C2D1. (B) Comparison of ADA levels at C2D1 according to the best response. ADA, atezolizumab anti-drug antibody; C2D1, cycle 2 day 1; CR, complete response; PR, partial response; SD, stable disease.

© Kim C, et al. *JAMA Oncol.* 2022.

eFigure 3. Bar charts showing the best response to therapy by ADA status

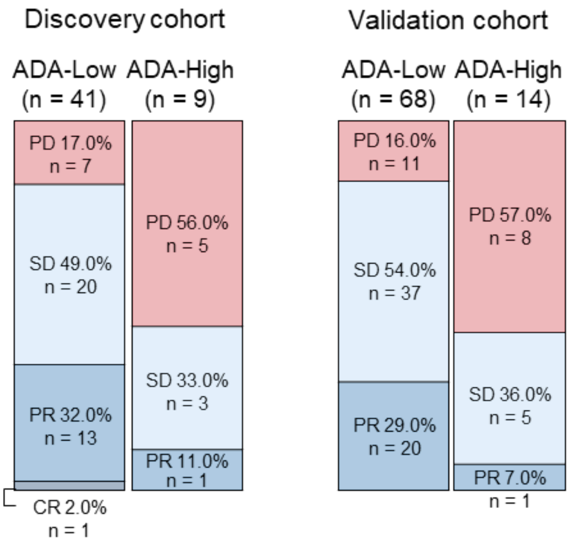

© Kim C, et al. *JAMA Oncol*. 2022.

eFigure 4. A time-dependent receiver operating characteristic curve for 12-months OS

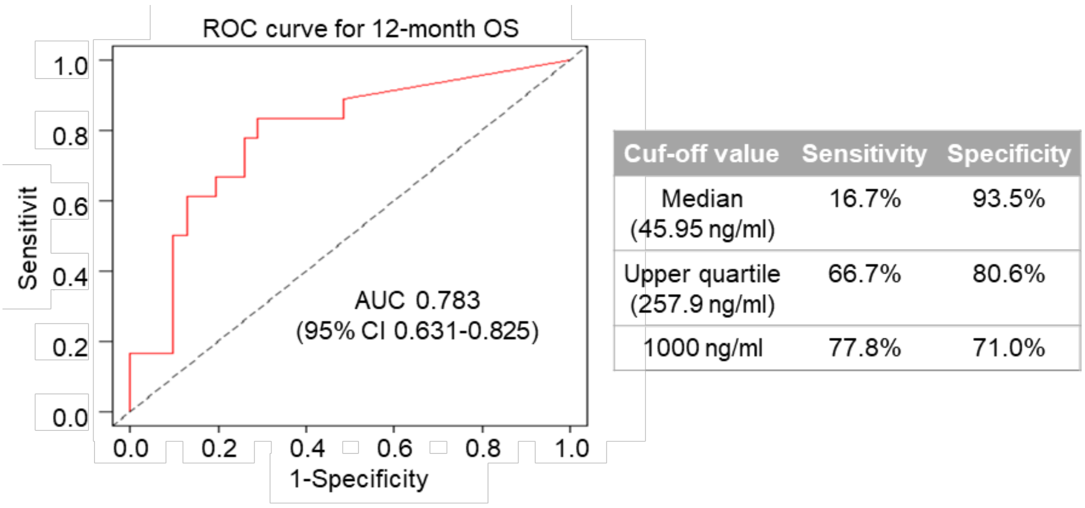

Cuf-off value	Sensitivity	Specificity
Median (45.95 ng/ml)	16.7%	93.5%
Upper quartile (257.9 ng/ml)	66.7%	80.6%
1000 ng/ml	77.8%	71.0%

© Kim C, et al. *JAMA Oncol*. 2022.

eFigure 5. Multivariable analysis of PFS and OS according to ADA status

A

Variable		n (%)	Multivariate HR (95% CI)	*P*
Age	>65 vs. ≤65	51 (38.6)<br>81 (61.4)	1.230<br>(0.768, 1.969)	0.389
Sex	Male vs. Female	111 (84.1)<br>21 (15.9)	1.110<br>(0.558, 2.205)	0.767
ECOG	1 vs. 0	61 (46.2)<br>71 (53.8)	1.110<br>(0.694, 1.776)	0.662
Child pugh score	A6 vs. A5	46 (34.8)<br>86 (65.2)	0.778<br>(0.457, 1.325)	0.356
AFP	>400 vs. ≤400	42 (31.8)<br>90 (68.2)	1.608<br>(0.955, 2.707)	0.074
MVI	Yes vs. No	55 (41.7)<br>77 (58.3)	1.467<br>(0.900, 2.389)	0.124
Extra-hepatic	Yes vs. No	74 (56.1)<br>58 (43.9)	1.159<br>(0.727, 1.848)	0.535
NLR			1.064<br>(0.987, 1.148)	0.105
ADA	≥1000 vs. <1000	23 (17.4)<br>109 (82.6)	2.137<br>(1.238, 3.689)	0.006

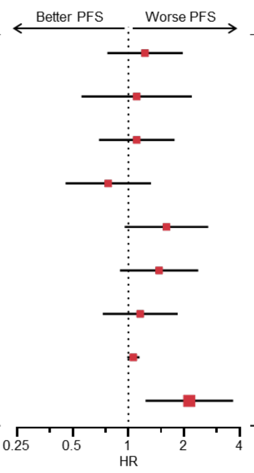

Variable		n (%)	Multivariate HR (95% CI)	*P*
Age	>65 vs. ≤65	51 (38.6)<br>81 (61.4)	0.769<br>(0.425, 1.393)	0.387
Sex	Male vs. Female	111 (84.1)<br>21 (15.9)	0.993<br>(0.436, 2.262)	0.987
ECOG	1 vs. 0	61 (46.2)<br>71 (53.8)	1.622<br>(0.912, 2.886)	0.100
Child pugh score	A6 vs. A5	46 (34.8)<br>86 (65.2)	1.768<br>(0.955, 3.274)	0.070
AFP	>400 vs. ≤400	42 (31.8)<br>90 (68.2)	1.944<br>(1.071, 3.528)	0.029
MVI	Yes vs. No	55 (41.7)<br>77 (58.3)	1.761<br>(0.976, 3.177)	0.060
Extra-hepatic	Yes vs. No	74 (56.1)<br>58 (43.9)	1.064<br>(0.602, 1.883)	0.830
NLR			1.054<br>(0.958, 1.159)	0.278
ADA	≥1000 vs. <1000	23 (17.4)<br>109 (82.6)	3.281<br>(1.773, 6.070)	<0.001

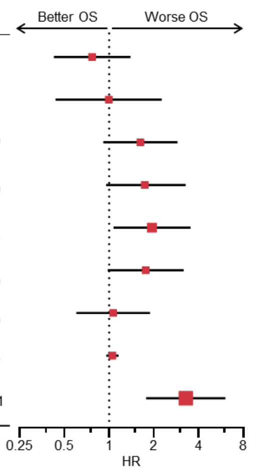

B

Cut-off value		Multivariate HR (95% CI)	*P*
ADA (ng/ml)	Median (45.95)	1.120<br>(0.711, 1.765)	0.625
	Upper quartile (257.9)	1.778<br>(1.059, 2.986)	0.030
	Upper 17 percentile (1000)	2.137<br>(1.238, 3.689)	0.006

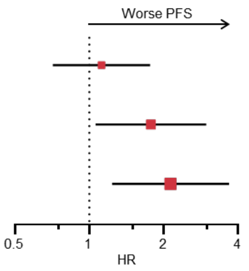

Cut-off value		Multivariate HR (95% CI)	*P*
ADA (ng/ml)	Median (45.95)	1.587<br>(0.899, 1.587)	0.111
	Upper quartile (257.9)	2.817<br>(1.570, 5.055)	0.001
	Upper 15 percentile (1000)	3.281<br>(1.773, 6.070)	<0.001

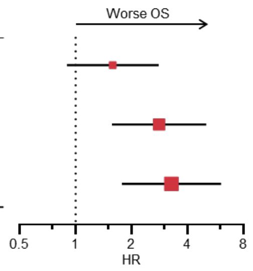

(A and B) Forest plots showing PFS (A) and OS (B) by age, sex, Eastern Cooperative Oncology Group (ECOG) performance status, Child-Pugh score, alpha-fetoprotein (AFP), macrovascular invasion (MVI), extrahepatic spread, neutrophil-lymphocyte ratio (NLR), and ADA status.

(C and D) Forest plots showing PFS (C) and OS (D) according to various cut-offs for ADA positivity. Multivariate analyses were conducted with Cox proportional hazard regression.

© Kim C, et al. *JAMA Oncol.* 2022.

eMethods

Study Approval

The study was approved by relevant institutional review boards (CHA Bundang Medical Center, CHA-2017-11-052, CHA-2017-11-054; Ulsan University Hospital, 2020-12-006; Haeundae Paik Hospital, 2020-12-019-001; St. Vincent's Hospital, VC16TISI0208). All patients signed written informed consent.

Treatments and evaluation

All patients in the HCC discovery and validation cohorts were treated with the combination of atezolizumab (1200 mg fixed dose) and bevacizumab (15 mg/kg) every 3 weeks. Dose interruptions or reductions were made according to the protocol of the IMbrave 150[1]. Treatment continued until patients experienced intolerable toxicity, disease progression, or consent withdrawal. The response was evaluated according to the RECIST 1.1. Response evaluation was performed every 6 or 9 weeks with computed tomography or magnetic resonance imaging.

Sample collection and ADA quantification

Blood samples were collected by venipuncture in Vacutainer tubes (BD Biosciences) before the first administration of atezolizumab (cycle 1 day 1, hereafter referred to as baseline) and before the second injection of atezolizumab (C2D1). Samples were left to coagulate, and serum was centrifuged at 1000 ×g for 5 min and stored at -80°C. Peripheral blood mononuclear cells were obtained by density gradient centrifugation with Ficoll-Paque PLUS (GE Healthcare). C2D1 blood samples that were drawn before 18 days or more than 24 days after the initiation of the first treatment were excluded from the analysis to ensure consistent timing.

Measurement of anti-atezolizumab antibodies and atezolizumab

The concentrations of anti-atezolizumab antibodies were measured using a enzyme-linked immunosorbent assay (ELISA) (KBI2027 ver 4.0, KRISHGEN BioSystems) according to the manufacturer's instructions. In brief, samples were incubated for 120 min at 37°C in a microwell pre-coated with atezolizumab. After several washings, horseradish peroxidase–conjugated atezolizumab was added and the plate incubated for 60 min at 37°C. After another washing, TMB substrate solution was added and the plate incubated for 30 min at 37°C in the dark. The absorbance was measured at 450 nm using a microplate reader (BioTek). The concentration of serum atezolizumab was measured using a commercial ELISA assay (KBI1027, KRISHGEN BioSystems).

Cytokine secretion assays and flow cytometry

To assess cytokine secretion, peripheral blood mononuclear cells were stimulated with plate-bound anti-CD3 antibody (1 ug/ml). After 4 hours, brefeldin A (eBioscience) and monensin (eBioscience) were added to the culture. After 20 hours, activated cells were harvested. To investigate proliferation capacity of $CD8^+$ T cells, cryopreserved peripheral blood mononuclear cells were used after thawing. Before antibody staining, dead cells were excluded by Fixable Viability Dye eFluorTM 780 (eBioscience) on ice for 30 min, followed by treatment with the human Fc receptor-binding inhibitor (eBioscience) for 15 min at room temperature. Surface proteins were stained with fluorochrome-conjugated antibodies, anti-human CD3 antibody (clone SK7, eBioscience), anti-human CD4 antibody (clone RPA-T4, BioLegend), and anti-human CD8 antibody (clone RPA-T8, eBioscience) on ice for 30 min. For intracellular staining, cells were fixed and permeabilized with FoxP3 staining buffer kit (Thermo Fisher Scientific) and stained with fluorochrome-conjugated antibodies, anti-human Ki-67 antibody (clone Ki-67, BioLegend), anti-human interferon-γ antibody (clone B27, BD Bioscience), and

© Kim C, et al. *JAMA Oncol.* 2022.

anti-human tumor necrosis factor-α antibody (clone Mab11, BD Bioscience). Flow cytometry was performed using a CytoFLEX flow cytometer (Beckman Coulter), and the resulting statistics were analyzed using FlowJo software (Tree Star Inc., Ashland, OR, USA).

Statistical analysis

The independent sample t-test was used to compare cytokine production between the ADA-high and ADA-low groups. The paired sample t-test was used to compare ADA levels and T cell proliferation between baseline and cycle 2 day 1. Analysis of variance was used to compare ADA levels according to treatment responses and to compare atezolizumab concentration according to ADA levels. Pearson's correlation was used to relate serum ADA and atezolizumab. OS was defined as the time from the initiation of treatment until death from any cause. Progression-free survival (PFS) was defined as the time between the initiation of treatment and the progression of the disease or death from any cause. Survival outcomes were analyzed using the Kaplan–Meier method, and the subgroups were compared using the log-rank test. Univariate and multivariate analyses of OS and PFS were performed using the Cox proportional hazards model. Statistical significance was defined as two-sided $P < 0.05$. The time-dependent ROC curve was analyzed using R version 4.0. Other statistical analyses were performed with SPSS (IBM) version 18.0.

© Kim C, et al. *JAMA Oncol*. 2022.
